# Disrupted topological organization of the default mode network in mild cognitive impairment with subsyndromal depression: A graph theoretical analysis

**DOI:** 10.1111/cns.14547

**Published:** 2023-12-17

**Authors:** Yang Du, Jing Nie, Jian‐Ye Zhang, Yuan Fang, Wen‐Jing Wei, Jing‐Hua Wang, Shao‐Wei Zhang, Jin‐Hong Wang, Xia Li

**Affiliations:** ^1^ Department of Geriatric Psychiatry, Shanghai Mental Health Center Shanghai Jiao Tong University School of Medicine Shanghai China; ^2^ Alzheimer's Disease and Related Disorders Center Shanghai Jiao Tong University Shanghai China; ^3^ Shanghai Key Laboratory of Psychotic Disorders, Shanghai Mental Health Center Shanghai Jiao Tong University School of Medicine Shanghai China

**Keywords:** default mode network, depression, graph theory, mild cognitive impairment, small‐worldness

## Abstract

**Aims:**

Subsyndromal depression (SSD) is common in mild cognitive impairment (MCI). However, the neural mechanisms underlying MCI with SSD (MCID) are unclear. The default mode network (DMN) is associated with cognitive processes and depressive symptoms. Therefore, we aimed to explore the topological organization of the DMN in patients with MCID.

**Methods:**

Forty‐two MCID patients, 34 MCI patients without SSD (MCIND), and 36 matched healthy controls (HCs) were enrolled. The resting‐state functional connectivity of the DMN of the participants was analyzed using a graph theoretical approach. Correlation analyses of network topological metrics, depressive symptoms, and cognitive function were conducted. Moreover, support vector machine (SVM) models were constructed based on topological metrics to distinguish MCID from MCIND. Finally, we used 10 repeats of 5‐fold cross‐validation for performance verification.

**Results:**

We found that the global efficiency and nodal efficiency of the left anterior medial prefrontal cortex (aMPFC) of the MCID group were significantly lower than the MCIND group. Moreover, small‐worldness and global efficiency were negatively correlated with depressive symptoms in MCID, and the nodal efficiency of the left lateral temporal cortex and left aMPFC was positively correlated with cognitive function in MCID. In cross‐validation, the SVM model had an accuracy of 0.83 [95% CI 0.79–0.87], a sensitivity of 0.88 [95% CI 0.86–0.90], a specificity of 0.75 [95% CI 0.72–0.78] and an area under the curve of 0.88 [95% CI 0.85–0.91].

**Conclusions:**

The coexistence of MCI and SSD was associated with the greatest disrupted topological organization of the DMN. The network topological metrics could identify MCID and serve as biomarkers of different clinical phenotypic presentations of MCI.

## Graphical abstract

The global efficiency and nodal efficiency were significantly lower in mild cognitive impairment with SSD (MCID), and the accuracy of the support vector machine model was 0.83 for distinguishing MCID from MCIND. MCID is correlated to the greatest disrupted topological organization, which may serve as biomarkers of different MCI subgroups.

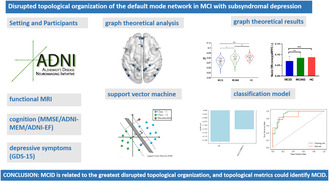

## 1 INTRODUCTION

Mild cognitive impairment (MCI), a stage between normal aging and early Alzheimer's disease (AD), is characterized by objective cognitive impairment with preserved activities of daily living.^1^ Subthreshold depression (SSD) was commonly associated with MCI, and the prevalence of SSD was higher in MCI patients than in normal individuals.^2^ SSD is typically defined as the frequency or intensity of depressive symptoms that do not meet the diagnostic criteria for depression.^3^ Previous studies suggest that the clinical significance of depressive symptoms does not depend on crossing the diagnostic threshold of depression, and SSD exists on a continuum with clinical depression.^4^ Of note, the prevalence of SSD is higher than clinical depression in older adults and is also correlated with MCI progression.^5^ Some evidence indicates severe functional disability and accelerated cognitive decline are associated with SSD in MCI patients.^6^ Despite the high prevalence and clinical importance of SSD in MCI patients, the neurobiological mechanisms of SSD in MCI remain largely unclear.

With the rapid advances in neuroimaging technology, exploring depressive symptomatology in MCI has been strongly supported. Graph theoretical analysis is a technique widely employed in neurodegenerative diseases to quantify brain connectivity and gain insights into the topological organization of biochemical networks.^7^, ^8^ Emerging evidence suggests that the default mode network (DMN) is one of the most prominent large‐scale intrinsic networks involved in episodic memory retrieval and emotion regulation processes, acting as potentially valuable biomarkers of depression and MCI.^9^, ^10^ Several studies found that MCI patients showed disrupted topological changes of the DMN compared to healthy controls,^11^, ^12^ suggesting that the DMN has essential implications for the pathophysiologic mechanisms of MCI. Previous studies found that the abnormal global topology of the DMN in depression patients was correlated with cognitive performance,^13^ indicating that the topological organization of the DMN contributes to cognitive deficits in depression. However, no studies have been conducted to explore the topological organization of MCI patients with SSD (MCID). Moreover, one graph theoretical analysis found that MCI patients with late‐life depression showed greater functional brain network topology disruptions than MCI patients without depression.^14^ However, the above study suggested whole‐brain functional network disturbances in MCI patients with depression without exploring the sub‐brain functional network of the DMN topological alterations. Exploring the topological organization of the DMN in MCID patients can better understand the presence of SSD in MCI patients, facilitating individualized treatment and prevention strategies.

Our study aimed to explore the network topology of the DMN in MCID patients, MCI patients without SSD (MCIND), and healthy controls (HCs) using graph theoretical analyses. We investigated (1) global network metrics, including the clustering coefficient (*C*_p_), shortest path length (*L*_p_), normalized clustering coefficient (*γ*), normalized shortest path length (*λ*), small‐worldness (*σ*), global efficiency (*E*_g_), and local efficiency (*E*_loc_); (2) regional network metrics, including degree centrality, betweenness centrality, and nodal efficiency (*E*_nod_); (3) correlations of abnormal network metrics with clinical symptoms such as depressive symptoms and cognitive function; and (4) construction of a classification model based on abnormal network metrics for distinguishing MCID from MCIND.

## 2 MATERIALS AND METHODS

### 2.1 Participants

The data used in this article were obtained from the Alzheimer's Disease Neuroimaging Initiative (ADNI) database (www.loni.ucla.edu/ADNI). We recruited 42 patients with MCID, 34 patients with MCIND, and 36 healthy control subjects (HCs) matched for age, sex, and education level. The use of the ADNI data was approved by the institutional review board at each site, and all the participants provided their written permission. Moreover, Mini‐Mental State Examination (MMSE) scores were used to evaluate participants' global cognition.^15^ The composite scores of memory (ADNI‐MEM) and executive function (ADNI‐EF) were generated by the ADNI neuropsychological battery to reflect memory and executive functions, respectively.^16^ The 15‐item Geriatric Depression Scale (GDS‐15) was used to assess depressive symptoms.^17^ In studies using the GDS‐15 to assess depressive symptoms in older adults, scores higher than 5 indicated the presence of clinical depression.^18^ Furthermore, participants' demographic information, medical history, baseline symptoms, and assessment scale scores were obtained from the ADNI database.

The diagnosis of MCI was based on the National Institute of Neurological and Communicative Disorders and Stroke and the AD and Related Disorders Association (NINCDS‐ADRDA) criteria.^19^ SSD was considered to have GDS‐15 scores greater than 0 and less than 6, with depressive symptoms present, but did not meet the full criteria for major depression according to the fifth edition of the Diagnostic and Statistical Manual of Mental Disorders (DSM‐5).^20^ The inclusion criteria for MCID were: (1) age between 55 and 90 years; (2) MMSE score between 24 and 30^21^; (3) a Clinical Dementia Rating scale (CDR) score of 0.5^22^; and (4) GDS‐15 score greater than 0 and less than 6^23^; and they did not meet the diagnostic criteria for major depressive disorder. Moreover, patients in the MCIND group included (1) those aged between 55 and 90 years; (2) those with MMSE scores between 24 and 30; (3) those with a CDR score of 0.5; and (4) those with a GDS‐15 score of 0. Additionally, health control (HC) subjects were included if they (1) were aged between 55 and 90 years; (2) had MMSE scores equal to or greater than 27; (3) had a CDR score of 0; and (4) had a GDS‐15 score of 0. The exclusion criteria for all subjects were as follows: (1) modified Hachinski ischemic score (HIS) greater than 4^24^; (2) significant neurological or psychiatric illness; (3) current use of antidepressants; and (4) inability to undergo structural magnetic resonance imaging (MRI) and functional MRI. Notably, all subjects were right‐handed.

### 2.2 Data acquisition and preprocessing

All the images were obtained from the ADNI database. T1‐weighted structural images were acquired using the magnetization‐prepared rapid gradient echo (MPRAGE) sequence with the following parameters: repetition time (TR) = 6.8 ms, echo time (TE) = 3.2 ms, flip angle = 9°, slice thickness = 1.2 mm, voxel size = 1 × 1 × 1.2 mm^3^, and matrix = 256 × 256 mm^2^. For the functional images, the following parameters were used: TR = 3000 ms, TE = 30 ms, flip angle = 80°, slice thickness = 3.3 mm, voxel size = 3.3 × 3.3 × 3.3 mm^3^, matrix = 64 × 64, and 140 time points in each run.

Imaging preprocessing was performed using the Data Processing Assistant for Resting‐State fMRI (DPARSF, http://www.restfmri.net/forum/dparsf).^25^ The first 10 images were discarded for magnetization equilibrium. Then, slice timing and head motion correction were performed for the remaining 130 images. To minimize the effect of head motion, we excluded the participants whose maximal head movement translation exceeded 2 mm, whose mean framewise displacement (FD) was more than 0.2 mm, or whose rotation was more than 2°. Consequently, no participant was excluded due to excessive head motion. Next, the images were normalized to standard Montreal Neurological Institute (MNI) space using the DARTEL algorithm, and each voxel was spatially resampled to a voxel size of 3 × 3 × 3 mm^3^. The cerebrospinal fluid signal, white matter, and Friston‐24 motion parameters were considered nuisance covariates. Subsequently, functional images were spatially smoothed with a 6 mm full‐width half‐maximum (FWHM) isotropic Gaussian kernel. Finally, bandpass filtering (0.01–0.08 Hz) was conducted to decrease the effect of systematic drift and high‐frequency noise.

### 2.3 Network construction and analysis

All network analyses in this study were performed using the Graph Theoretical Network Analysis (GRETNA) toolbox (http://www.nitrc.org/projects/gretna/). First, the DMN comprises the anterior medial prefrontal cortex (aMPFC), posterior cingulate cortex (PCC), hippocampal formation, ventral medial prefrontal cortex (vMPFC), the dMPFC, lateral temporal cortex (LTC), and other areas.^26^ More details about a specific set of 20 regions of interest (ROIs) of the DMN and the corresponding MNI coordinates can be found in Table 1. Moreover, we calculated the mean time series for all voxels within the ROI of the DMN using spherical seeds based on the MNI coordinate system. For each subject, the Pearson correlation coefficients between the mean time series of all pairs of 20 regions were computed, yielding a 20 × 20 correlation matrix for the DMN. We threshold at a wide range of sparsity levels according to the following criteria: (1) on the basis that there are no isolated nodes in the human brain, the average degree over all nodes of each network was larger than the result of the function gretna_get_rmax(rand(*N*, *N*)), where *N* = 20, denoting the number of nodes; and (2) the small‐worldness of the networks was larger than 1.1 for all subjects.^27^ In this study, the range of values generated for the threshold, from 0.16 to 0.34 with an interval of 0.01, was applied to all matrices.

**TABLE 1:** Coordinates of 20 predefined regions of interest.

Regions	Abbreviation	Brodmann areas	MNI (*x*, *y*, *z*)		
Anterior medial prefrontal cortex	aMPFC.L aMPFC.R	10, 32	−6	52	−2
			6	52	−2
Dorsal medial prefrontal cortex	dMPFC	9, 32	0	52	26
Ventral medial prefrontal cortex	vMPFC	11, 24, 25, 36	0	26	−18
Posterior cingulate cortex	PCC.L PCC.R	23, 31	−8	−56	26
			8	−56	26
Temporal parietal junction	TPJ.L TPJ.R	40,39	−54	−54	28
			54	−54	28
Lateral temporal cortex	LTC.L LTC.R	21, 22	−60	−24	−18
			60	−24	−18
Temporal pole	TempP.L TempP.R	21	−50	14	−40
			50	14	−40
Posterior inferior parietal lobule	pIPL.L pIPL.R	39	−44	−74	32
			44	−74	32
Retrosplenial cortex	Rsp.L Rsp.R	29, 30, 19	−14	−52	8
			14	−52	8
Parahippocampal cortex	PHC.L PHC.R	20, 36, 19	−28	−40	−12
			28	−40	−12
Hippocampal formation	HF.L HF.R	20, 36	−22	−20	−26
			22	−20	−26

Abbreviation: MNI, Montreal Neurological Institute.

The global network metrics included *C*_p_, *γ*, *L*_p_, *λ*, *σ*, *E*_g_, and *E*_loc_. *C*_p_ measures the degree to which neighboring brain regions are connected, and *L*_p_ quantifies the mean distance between the areas along the shortest path.^28^ Moreover, the small‐worldness of a network, an optimal balance between the segregation and integration of information processing procedures, is defined as the ratio of the normalized clustering coefficient (*γ* = *C*_real_/*C*_random_) and the normalized shortest path length (*λ* = *L*_real_/*L*_random_).^29^ According to the definition, the small‐world network should satisfy the following conditions: *γ* > 1 and *λ* ≈ 1 or *σ* (=*γ*/*λ*) > 1.^30^ Moreover, *E*_glob_ measures the efficiency of the delivery of parallel messages on a global scale, while *E*_loc_ measures the efficiency of the delivery of information at the level of a single node.^31^ The regional network metric of nodal efficiency (*E*_nod_) characterizes the efficiency of parallel information transfer.^32^ Finally, we calculated the area under the curve (AUC) for each global and regional metric over a range of densities instead of selecting a single sparsity threshold, which delivered a summarized measure for the topological characterization independent of the effect of choosing a single threshold.^33^

### 2.4 Statistical analysis

We tested for normality using the Shapiro–Wilk test for all data. For data following the normal distribution, we used analysis of variance to compare the differences among the MCID, MCIND, and HC groups. Then, the sources of the differences among the means of the three groups were examined by the post hoc Fisher's least significant difference (LSD) test. For data that did not satisfy a normal distribution, we used the nonparametric Kruskal‐Wallis test to compare the differences among the three groups. We conducted chi‐square tests for gender characteristics in the MCID, MCIND, and HC groups. Moreover, nonparametric permutation tests^34^ with 10,000 permutations were used to determine significant between‐group differences in the AUC of all metrics controlling for age, sex, and education. Additionally, we used false discovery rate (FDR) correction for multiple comparisons,^35^ and the significance level was set at *p* < 0.05. To better localize specific brain regions with altered functional connectivity in MICD patients, we used a network‐based statistics (NBS) approach.^36^ Once differences between groups were found to be statistically significant, we explored the relationships between the abnormal network metrics of the DMN and clinical characteristics (MMSE, ADNI‐MEM, ADNI‐EF, and GDS scores) using a correlation analysis in each participant group separately while controlling for age, sex, and education. Considering that the clinical data and neuroimaging metrics may not be normally distributed, we chose either Pearson's correlation or Spearman's rank correlation test, depending on the normal distribution of the data.^37^ The significance level was set at *p* < 0.05 and corrected using the false discovery rate (FDR) method.

### 2.5 Classification analysis

First, patients with MCI were randomly divided into training and test datasets at proportions of 0.7 and 0.3. Second, the *t*‐test and Mann‐Whitney U test were used to select the features with significant differences (*p* < 0.05). Then, a 10‐fold cross‐validated least absolute shrinkage selection operator (LASSO) regression analysis is used to determine the most effective features in the training data, and the corresponding lambda values are selected with the minimum mean squared error (MSE) values.^38^ Support vector machine (SVM) algorithms were adopted to construct a model for MCID and MCIND. SVM is currently one of the most popular and mature machine learning algorithms for neuroimaging research.^39^ Moreover, we used 10 repeats of 5‐fold cross‐validation for model performance verification. Additionally, receiver operating characteristic (ROC) curves and the corresponding areas under the curve (AUCs) were used to evaluate the diagnostic capabilities of the model.

## 3 RESULTS

### 3.1 Demographic and clinical data

The demographic and clinical information are summarized in Table 2. The results of the Shapiro‐Wilk test showed that all demographic and clinical data followed a normal distribution (*p* > 0.05) except for the GDS scores (*p* < 0.001). We found no significant differences among the MCID, MCIND, and HC groups in age, sex, or education (*p* > 0.05). For clinical characteristics, the MMSE and ADNI‐MEM scores of participants in the MCID and MCIND groups were significantly lower compared to those of the HC group, while ADNI‐MEM scores were not significantly different among the three groups. Conversely, participants in the MCID group had significantly higher GDS scores than those in the other two groups. These results are summarized in Table 2.

**TABLE 2:** Demographic and clinical characteristics.

Characteristics	MCID (*N* = 42)	MCIND (*N* = 34)	HC (*N* = 36)	*p* value
Age (years)	74.11 ± 7.92	74.26 ± 5.13	74.32 ± 4.65	0.911
Sex (M/F)	22/18	17/15	20/16	0.978
Education (years)	16.55 ± 2.12	16.51 ± 2.35	16.49 ± 2.58	0.358
MMSE	26.30 ± 2.27	26.87 ± 2.24	28.83 ± 1.32	**0.00002^a^; 0.00039^b^**
ADNI‐MEM	0.30 ± 0.78	0.31 ± 0.71	1.04 ± 0.55	**0.00003^a^;0.00017^b^**
ADNI‐EF	0.35 ± 1.11	0.41 ± 0.96	0.67 ± 0.77	0.207
CDR	0.5	0.5	0	‐
GDS‐15	2.43 ± 1.41	0	0	**4.9E‐15^a^;1.1E‐15^c^**

*Note*: Data are presented as mean (M) ± standard deviation (SD).

Bold indicates statistical significant value (*p* < 0.001).

Abbreviations: ADNI‐EF, ADNI composite score for executive function (EF); ADNI‐MEM, Alzheimer's Disease Neuroimaging Initiative (ADNI) composite score for episodic memory (MEM); CDR, Clinical Dementia Rating Scale; GDS‐15, the 15 items of Geriatric Depression Screening Scale; HC, healthy controls; MCID, MCI patients with subthreshold depressive symptoms; MCIND, MCI patients without subthreshold depressive symptoms; MMSE, Mini Mental State Examination; a‐c: post hoc analysis following one‐way analysis of variance (a: HC vs. MCID, b: HC vs. MCIND, c: MCID vs. MCIND).

### 3.2 Global graph theoretical analyses

**FIGURE 1:**
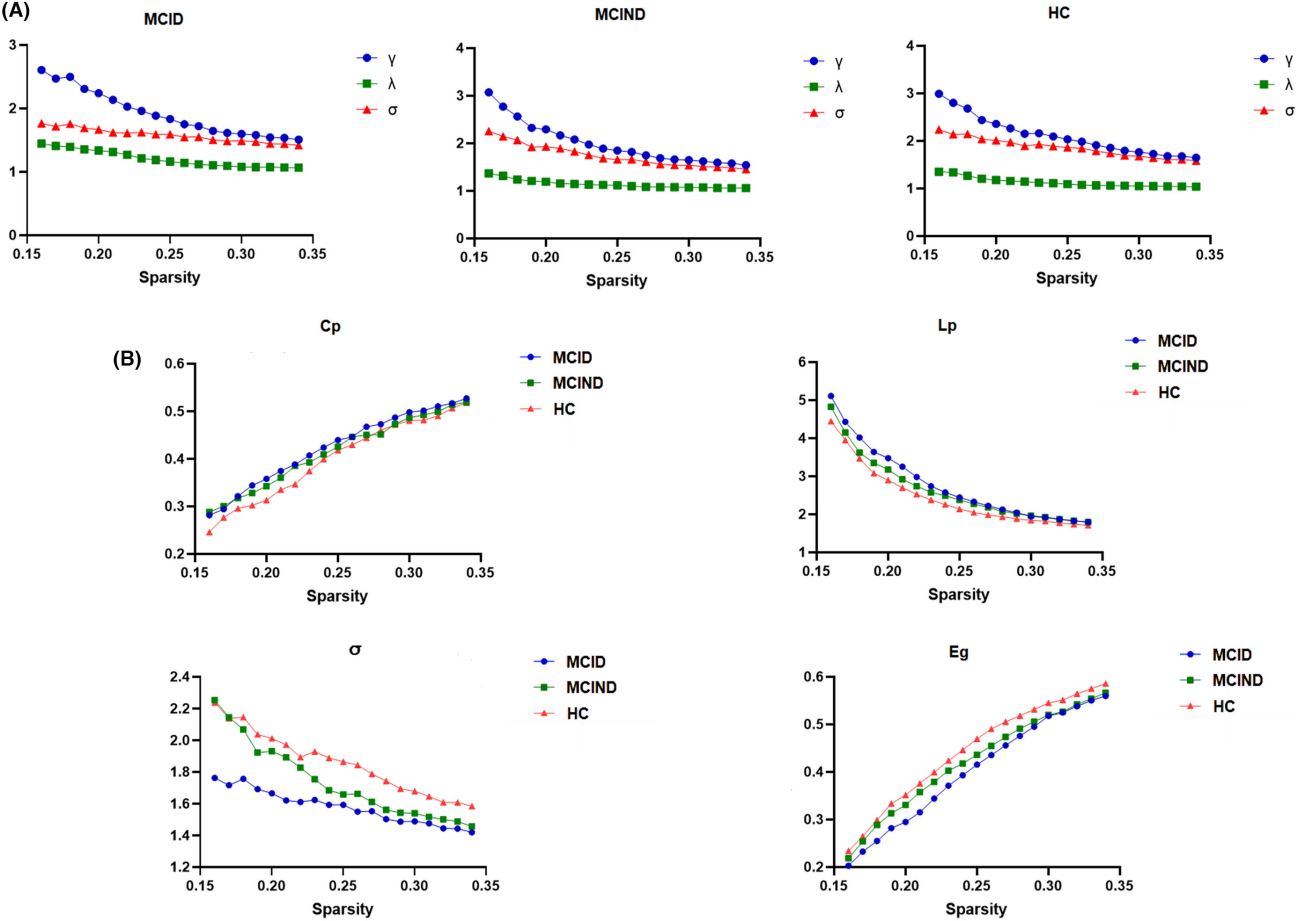
Small‐world properties of the DMN in MCID, MCIND, and HC groups (A). Global topographic metrics in MICD, MCIND, and HC groups with the selected range of sparsity threshold (B). *C*_p_, clustering coefficient; DMN, default‐mode network; *E*_g_, global efficiency; HC, healthy control; *L*_p_, shortest path length; MCID, MCI patients with subthreshold depressive symptoms; MCIND, MCI patients without subthreshold depression; *σ*, small‐worldness.

**FIGURE 2:**
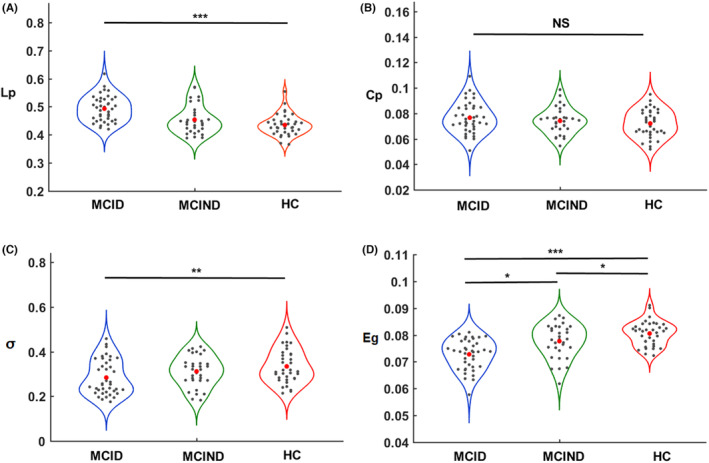
Differences in global network metrics of the DMN, including the *L*_p_ (A), *C*_p_ (B), *σ* (C), and *E*_g_ (D) between MICD, MICND, and HC groups. The values on the *Y*‐axis indicate the AUC of the graph metrics within the sparsity threshold. The red dots represent what the group means. AUC, the area under the curve. (*p < 0.05; **p < 0.01; ***p < 0.001).

MCID, MCIND, and HC subjects demonstrated typical small‐world properties within the defined thresholds. *λ* was close to 1, and γ and σ were greater than 1 (Figure 1A). Figure 1B shows the changes in global parameters, including *C*_p_, *L*_p_, *σ* and *E*_g_, in the three groups as a function of sparsity thresholds. The Shapiro–Wilk test showed that *σ* (*p* = 0.007) in the MCID group and *L*_p_ (*p* = 0.010) in the MCIND group did not satisfy the normal distribution, and *E*_g_ and *C*_p_ (*p* > 0.05) followed the normal distribution. Compared with the HC group, the MCID group showed significantly higher values in the *L*_p_ (*p*_MCID vs. HC_ < 0.001; Figure 2A). However, *C*_p_ had no significant between‐group difference (Figure 2B). Furthermore, the *σ* of the MCID group was significantly lower than that of the HC group (*p*_MCID vs. HC_ = 0.018; Figure 2C). In addition, significantly lower *E*_g_ was observed between the MCID and HC groups, the MCIND and HC groups, and the MCID and MCIND groups (*p*_MCID vs. HC_ < 0.001; *p*_MCIND vs. HC_ = 0.024; *p*_MCID vs. MCIND_ = 0.013; Figure 2D). No significant correlations were found between any global topological metrics and cognitive function.

### 3.3 Regional graph theoretical analyses

**FIGURE 3:**
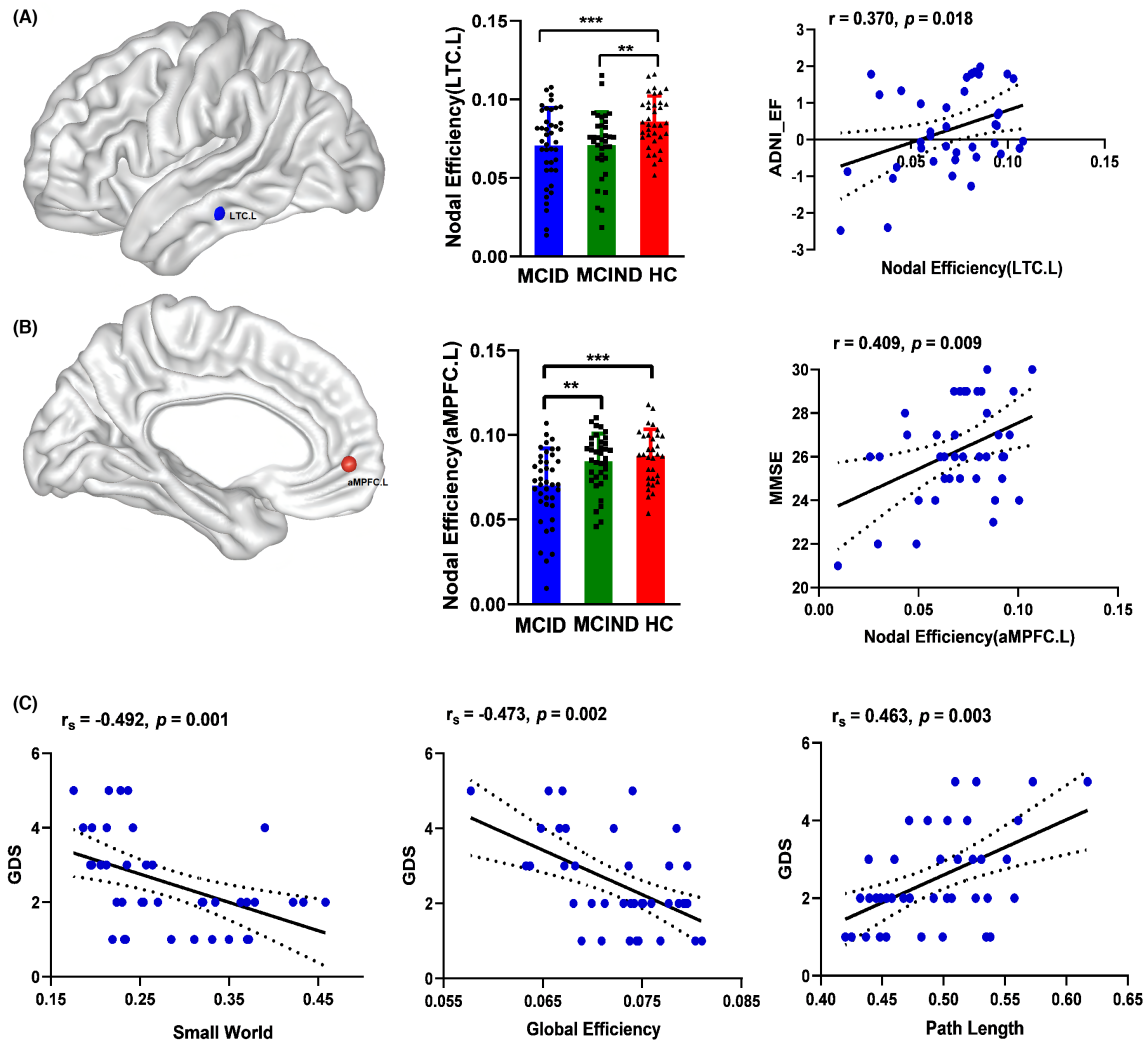
Brain regions show abnormal nodal efficiency of the DMN between MCID and MCIND patients and HCs. The lower *E*_nod_ of the LTC.L in MICD positively correlated with ADNI_EF scores (A). The lower *E*_nod_ of the aMPFC.L in MICD positively correlated with MMSE scores (B). Global network metrics correlated with depressive symptoms in MICD patients (C). ADNI‐EF, ADNI composite score for executive function (EF); aMPFC.L, the left anterior medial prefrontal cortex; *E*_nod_, nodal efficiency; LTC.L, the left lateral temporal cortex; MMSE, Mini‐Mental State Examination. *p < 0.05; **p < 0.01; ***p < 0.001.

The Shapiro‐Wilk test demonstrated that the regional metrics of the LTG and aMPFC followed a normal distribution. MCID patients and MCIND patients had significantly lower *E*_nod_ in the left LTC (LTC.L) relative to the HC group (Figure 3A, *p* < 0.05, FDR corrected). Moreover, the MCID group showed a significantly lower *E*_nod_ in the left aMPFC (aMPFC.L) compared with the MCIND and HC groups (Figure 3B, *p* < 0.05, FDR corrected). Moreover, the results of Pearson correlation analysis showed that the *E*_nod_ of LTC.L was positively correlated with the ADNI‐EF scores (*r* = 0.370, *p* = 0.018, FDR corrected) in MCID patients (Figure 3A). The *E*_nod_ of aMPFC.L was positively correlated with MMSE scores (*r* = 0.409, *p* = 0.009, FDR corrected) in the MCID group (Figure 3B). None of the topological measures were significantly associated with cognitive function in the MCIND and HC groups.

### 3.4 Relationships of topological metrics with depressive symptoms

Since GDS scores are not normally distributed data, we tested the relationship between global and regional topological metrics in the DMN and subthreshold depression measured by the GDS‐15 using Spearman correlation analysis. The Spearman coefficient is abbreviated as “*r*_s._” The small‐world property of the DMN network was negatively correlated with depressive symptoms (*r*_s_ = −0.492, *p* = 0.001, FDR corrected) in the MCID group (Figure 3C). The global efficiency of the DMN was also negatively correlated with depressive symptoms (*r*_s_ = −0.473, *p* = 0.002, FDR corrected) in the MCID group (Figure 3C). Likewise, a significant negative relationship existed between path length and depressive symptoms (*r*_s_ = 0.463, *p* = 0.003, FDR corrected) in MCID patients (Figure 3C). However, there was no significant correlation between other global and regional topological metrics and the severity of subthreshold depression.

### 3.5 Classification analysis results

**FIGURE 4:**
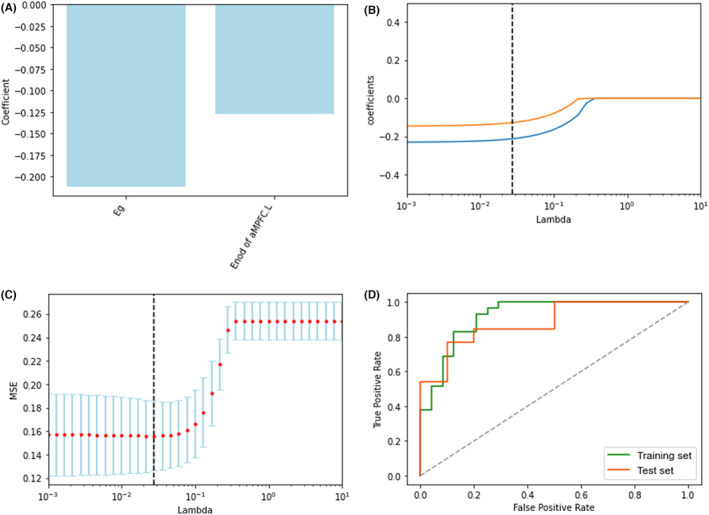
The selected metrics for distinguishing MCID from MCIND following LASSO regression (A), The coefficients‐lambda graph (B) and the MSE‐lambda graph (C) in the MCID and MCIND groups, the ROC curve of the training and test sets of the selected metrics for distinguishing MCID from MCIND (D). LASSO, least absolute shrinkage selection operator; MSE, mean squared error; ROC, receiver operating characteristic.

The LASSO regression model identified two topological metrics, including *E*_g_ and the *E*_nod_ of aMPFC.L for MCID and MCIND groups (Figure 4A). Meanwhile, the values of the coefficients and the corresponding lambda values, and the MSE values and the corresponding lambda values for the MCID and MCIND groups, are shown in Figure 4B,C. In the analysis between the MCID and MCIND patients, the accuracy, sensitivity, specificity, and AUC were 0.83, 0.84, 0.82, and 0.92 in the training set and 0.83, 0.86, 0.78, and 0.88 in the test set, respectively (Figure 4D). In cross‐validation, the SVM model with two topological metrics had an accuracy of 0.83 [95% CI 0.79–0.87], a sensitivity of 0.88 [95% CI 0.86–0.90], a specificity of 0.75 [95% CI 0.72–0.78], and an AUC of 0.88 [95% CI 0.85–0.91]. The results of the cross‐validation method suggested that the model has relatively good discrimination ability.

## 4 DISCUSSION

Our study found changes in the network topology of the DMN in MCI patients with SSD using graph network analysis. More specifically, the global metric of *E*_g_ was significantly lower in MCID patients than in MCIND patients. The L_p_ was significantly higher, and *E*_g_ and *σ* were significantly lower in MCID patients than in HCs. Furthermore, the global metrics of *σ* , *L*_p_, and *E*_g_ were correlated with depressive symptoms in MCID. For the regional metrics, the MCID group showed a significantly lower *E*_nod_ of the left aMPFC than the MCIND group. The *E*_nod_ of the left LTC and left aMPFC were significantly lower in MCID patients than in HCs. Additionally, the regional metrics of *E*_nod_ of the left LTC and left aMPFC were correlated with cognitive function in MCID. Moreover, we found that the classification model based on topological metrics can discriminate patients with MCID from MCIND with relatively successful diagnostic values. The findings may provide novel insights into the underlying pathophysiology of SSD in MCI.

In our study, *L*_p_ was significantly higher and *σ* and *E*_g_ were significantly lower within the DMN in the MCID group than in the HC group. The small‐world properties facilitate the description of a complex brain network, which is a balance between high segregation and integration, maximizing the information propagation efficiency.^40^ More precisely, functional segregation could be measured by *E*_loc_ and *C*_P_, and functional integration was characterized by *L*_p_ and *E*_g_.^41^ In our study, the higher *L*_p_ and lower *E*_g_ in the MCID group indicated functional integration disruption in the DMN, suggesting insufficient capability to combine specialized information from distributed brain regions in MCID patients. Generally, MCID patients had relatively reserved network segregation (no differential *C*_p_ and *E*_loc_) and decreased network integration (lower *E*_glob_ and higher *L*_p_), leading to a weaker small‐worldness (lower *σ*). It may be the combined result of neurodegeneration and abnormalities in the emotional circuitry. These findings suggested that MCID patients exhibit disturbances in the functional integration of the DMN, resulting in MCID patients who may not be able to coordinate cognitive resources properly.

Moreover, *E*_g_ was significantly lower in the DMN of patients in the MCID group than in participants in the MCIND group. A study revealed that MCI patients comorbid with depression exhibited the greatest disruptions in the functional network integration of diminished *E*_g_ compared with MCI patients and depression patients.^14^ Combined with our findings, MCI patients, even those with comorbid SSD rather than depression, also demonstrated the greatest disruptions in functional integration measures of reduced *E*_g_ relative to MCIND and HC groups. Evidence has shown that SSD is not fundamentally different from the current definition of depression and lies on a continuum with more severe forms of depressive episodes.^42^ Our study of MCI patients with SSD and a previous study of MCI patients with depression found similar changes in graph theory measures, supporting the fact that there is a continuum of depressive symptoms. In general, the findings provide novel evidence that MCID leads to a severely disrupted functional network organization of the DMN. Therefore, the global functional network metric of *E*_g_ may serve as a promising biomarker of different clinical phenotypic presentations of MCI, suggesting that MCID may be a specialized subtype of MCI with small‐world properties that differ from MCIND.

Moreover, we found that global network metrics, including *σ*, *E*_g_, and *L*_p_ were associated with subthreshold depressive symptoms measured by GDS‐15 scores in the MCID group. The findings suggest that more disrupted topological metrics in MCID patients are associated with more severe depressive symptoms. A previous study showed that the network metrics of *E*_g_ are significantly correlated with depressive symptoms in MCI patients with comorbid depression.^14^ However, few studies have focused on the relationship between topological metrics and the severity of SSD in MCID patients. Our study found that SSD was associated with *E*_g_ in MCID patients, similar to the above research, and may be consistent with the concept of a continuum of depression. The findings suggest that the regulation of the balanced state of functional integration and segregation of small‐world properties may lead to an improvement in depressive symptoms. In summary, these global network metrics may be useful biomarkers for identifying and assessing depressive symptoms in patients with MCID.

We observed a lower *E*_nod_ of the left LTC in the MCID and MCIND groups than in the HC group. The LTC belongs to the dorsal medial prefrontal cortex (dMPFC) subsystem, which preferentially engages in self‐referential judgments about present situations or mental states.^43^ One study that selected the left LTC as the targeted cortical region for anodal transcranial direct current stimulation (tDCS) found enhanced global cognition and executive function in patients with AD, suggesting that the left LTC was associated with cognitive function, particularly executive function.^44^ Moreover, the evidence indicated that the LTC is involved as an integral part of the cognitive network processing executive function.^45^ In our study, we observed that the *E*_nod_ of the left LTC was positively correlated with executive function measured by ADNI‐EF scores in the MCID group, highlighting the role of the left LTC in executive function. In summary, our results revealed that MCI, regardless of SSD status, significantly disrupted the nodal efficiency of the left LTC, providing a neural basis for the left LTC as a therapeutic target for MCI patients.

Additionally, the MCID group showed a lower *E*_nod_ of the left aMPFC than the MCIND and HC groups. The aMPFC serves as the core hub of the DMN, engaging in processes such as personal significance, introspection about one's mental states, and evoking emotion.^46^ Moreover, the *E*_nod_ of the left aMPFC is another metric of the selected features to distinguish MCI and MCIND, suggesting that the *E*_nod_ of the left aMPFC is a regional topological measure of identifying the presence or absence of SSD in MCI patients. Several studies have shown that the aMPFC is a crucial brain area in depressive symptomatology and is involved in the underlying pathology of depression,^47^ which could support our findings to some extent. Recent studies have shown that the aMPFC processes the emotional and cognitive functions required for adequately handling episodic and fear memory.^48^ In our study, the *E*_nod_ of the left aMPFC positively correlated with global cognition measured by MMSE in the MCID group, which is compatible with the role of the aMPFC in the development of cognitive impairments. The findings suggested that the left aMPFC may serve as a biomarker of different clinical phenotypic presentations of MCI.

To our knowledge, this study is the first to construct a classification model of topological metrics for MCID and MCIND patients. This model reveals relatively good accuracy and sensitivity, with relatively high discrimination power. A study showed distinct patterns of cognitive deficit in MCID and MCIND groups, suggesting the heterogeneity of MCI subgroups.^49^ Moreover, a study showed increased gray matter in the middle cingulate cortex accompanied by increased functional connectivity with the right parahippocampus in the MCID group compared to the MCIND group, providing neuroimaging evidence for the heterogeneity of MCI subgroups.^50^ Previous studies have shown that resting‐state functional connectivity can discriminate between mild cognitive impairment and healthy individuals with an accuracy of 73.49%.^51^ In the current study, topological metrics of the DMN were selected, global efficiency and nodal efficiency values of the left aMPFC survived after LASSO regression, and then a classification model with relatively high sensitivity was proposed. Our findings suggested that the pathophysiological mechanism of MCID was closely associated with the re‐organization of the DMN, providing key insights into the underlying pathophysiology of SSD in MCI. Moreover, disrupted topological metrics of the DMN could identify patients with MCID from MCIND and can be valid biomarkers to identify MCID patients and MCIND patients, which has great potential for personalized clinical application.

There were some limitations in this study. First, our study performed a graphical analysis using resting‐state fMRI of MCID, MCIND patients, and HCs. Evidence suggests that white matter (WM) damage is associated with depressive symptoms in MCI patients^52^; therefore, further studies on WM are warranted in future studies. Second, while we excluded the minority of MCID patients who were undergoing antidepressant therapy, most MCI patients were receiving pharmacological treatment to improve cognition, which may interfere with brain function. There is a future need to focus on this group in the preclinical phase of AD earlier than MCI with depressive symptoms and not receiving pharmacological treatment. Moreover, this study was cross‐sectional, and future longitudinal studies would be more beneficial for observing the cognitive function prognosis of MCI with or without SSD. Additionally, there are no standardized diagnostic criteria or screening tools for subthreshold depression, which leads to possible heterogeneity among relevant studies. Future research should agree on an operational definition of subthreshold depression. Finally, the sample size was limited. Future studies with a large sample size are necessary to validate our findings further.

Our findings provide evidence that the coexistence of SSD and MCI had the greatest disruptions of the DMN in topological metrics. Moreover, abnormal topological metrics not only correlate with cognitive function and depressive symptoms but also provide relatively good discrimination between MCID and MCIND. The findings may facilitate clarification of the underlying mechanisms of SSD and provide promising neuroimaging biomarkers of different clinical phenotypic presentations of MCI.

## CONFLICT OF INTEREST STATEMENT

The authors report no conflicts of interest.

## Data Availability

The use of the ADNI data was approved by the institutional review board at each site, and all the participants provided their written permission. For up‐to‐date information, see www.adni‐info.org. The data that support the findings of this study are openly available in Alzheimer’s Disease Neuroimaging Initiative at https://adni.loni.usc.edu/, reference number http://www.loni.ucla.edu/ADNI.
